# Changes in awareness and knowledge concerning mother-to-child infections among Japanese pregnant women between 2012 and 2018

**DOI:** 10.1371/journal.pone.0244945

**Published:** 2021-01-06

**Authors:** Shutaro Suga, Kazumichi Fujioka, Ruka Nakasone, Shinya Abe, Sachiyo Fukushima, Mariko Ashina, Kosuke Nishida, Kandai Nozu, Kazumoto Iijima, Kenji Tanimura, Hideto Yamada

**Affiliations:** 1 Department of Pediatrics, Kobe University Graduate School of Medicine, Kobe, Japan; 2 Department of Pediatrics, School of Medicine, University of Occupational and Environmental Health, Kitakyushu, Japan; 3 Department of Obstetrics and Gynecology, Kobe University Graduate School of Medicine, Kobe, Japan; Centre de Recherche en Cancerologie de Lyon, FRANCE

## Abstract

This study aimed to investigate the long-term changes in awareness of and knowledge about mother-to-child infections across 6 years in Japan. A questionnaire survey was conducted at our facility from October 2012 to January 2018, and the study periods were divided into 4 phases comprising 16 months each. A multiple-choice questionnaire assessed participants’ awareness of the following 13 pathogens of mother-to-child infections: cytomegalovirus (CMV), *Toxoplasma gondii* (*T*. *gondii*), hepatitis B virus, rubella virus, herpes simplex virus, parvovirus B19, hepatitis C virus, human immunodeficiency virus, human T cell leukemia virus type-1, measles virus, varicella-zoster virus, *Chlamydia trachomatis*, and *Treponema pallidum*. For the selected four pathogens (i.e., CMV, rubella virus, *T*. *gondii*, and parvovirus B19), the questionnaire also evaluated participants’ knowledge of transmission routes, the most susceptible time of infection that could yield severe fetal disease during pregnancy, the maximum frequency of fetal infection in cases of maternal infection, and methods to prevent maternal infection. In total, 1433 pregnant Japanese women were included in this study. There was no secular change in awareness of the pathogens concerning mother-to-child infections over time, and we also clarified that the detailed knowledge of the four pathogens of typical mother-to-child infections did not improve. Since knowledge about methods to prevent maternal infection is still insufficient for all pathogens, further advocacy is required to prevent mother-to-child infections.

## Introduction

Congenital infection is a common cause of neonatal morbidity and mortality. For example, the estimated incidence of the congenital cytomegalovirus (CMV) infection in Japan was reported to be 30 per 10,000 births between 2008 and 2010 [1], which is more frequent in comparison to trisomy 21 during the same period (i.e., 22 per 10,000 births) [2]. Meanwhile, in a nationwide survey investigating the incidence of congenital infections, Yamada et al. clarified the reported incidence of congenital infections among 34 cases with CMV and 1 with *Toxoplasma gondii* (*T*. *gondii*) from 2714 obstetric facilities in Japan. Based on these findings, they determined that many neonates with congenital infections of CMV or *T*. *gondii* may be undiagnosed during the pre- and post-natal periods in obstetric facilities [3].

Although vaccination before pregnancy is the most effective strategy for preventing mother-to-child transmission of diseases for some acute infections such as rubella and varicella, several major pathogens of congenital infections, such as CMV and *T*. *gondii*, lack effective vaccines. For mother-to-child transmitted infections that do not have effective vaccines, individual knowledge does directly link to the prevention of congenital infections. With regard to congenital CMV infections, the most common pathogens of the congenital infections with no effective vaccine, it is highlighted that the best prevention is to help pregnant women to be fully aware of basic hygiene practices [4]. Furthermore, Revello et al. reported that the infection rate was significantly lower in the group that received the serological test and information relating to CMV during pregnancy, compared to the group that did not receive the intervention (i.e., 1.2% vs 7.6%) [5]. A Belgian study also reported that the incidence of congenital toxoplasmosis significantly decreased after the introduction of intensive counseling for pregnant women regarding the disease [6].

In Japan, we previously conducted a single-center survey about awareness of and knowledge about mother-to-child infections in pregnant women between 2011 and 2012 [7]. In this questionnaire, awareness of 13 pathogens related to congenital infections and detailed knowledge concerning four representative pathogens that can be prevented through behavior change during pregnancy (i.e., CMV, rubella virus, *T*. *gondii*, and parvovirus B19) were inspected. The proportion of women aware of pathogens associated with the TORCH syndrome was as follows: rubella virus 76%, *Treponema pallidum* 69%, *T*. *gondii* 58%, parvovirus B19 28%, herpes simplex virus 27%, and CMV 18%. Notably, only 8% understood how CMV is transmitted, and only 12% were able to identify how parvovirus B19 was transmitted. Based on these outcomes, we concluded that awareness of and knowledge about both CMV and parvovirus B19 infection were insufficient among Japanese pregnant women.

To date, however, there has been no report regarding long-term changes in awareness of and knowledge about mother-to-child infections. In addition, there have been several historical changes/movements that have occurred in Japanese society since the first survey. From the perspective of infectious disease epidemics in Japan, a rubella epidemic occurred between 2012 and 2013, which caused approximately 17,000 rubella cases and led to 45 infants developing congenital rubella syndrome [8]. In response to this rubella epidemic, the Japanese government subsequently recommended voluntary vaccination or rubella antibody testing for young women who were planning to conceive and for adult men, children, and other persons in potential contact with pregnant women at home [9]. From a vaccine policy perspective in Japan, the varicella vaccine for 1-year-old infants was added to the national routine immunization program in November 2014 [10]. Subsequently, the hepatitis B vaccine for infants under 1 year old was added to the national routine immunization program in October, 2016 [11]. Thus, we hypothesized that these historical events that took place between 2012 to 2018 may have affected awareness and knowledge concerning mother-to-child infections among Japanese pregnant women.

Therefore, we studied changes in the awareness and knowledge of pregnant women who visited Kobe University Hospital across 6 years.

## Methods

### Study design

**Fig 1:**
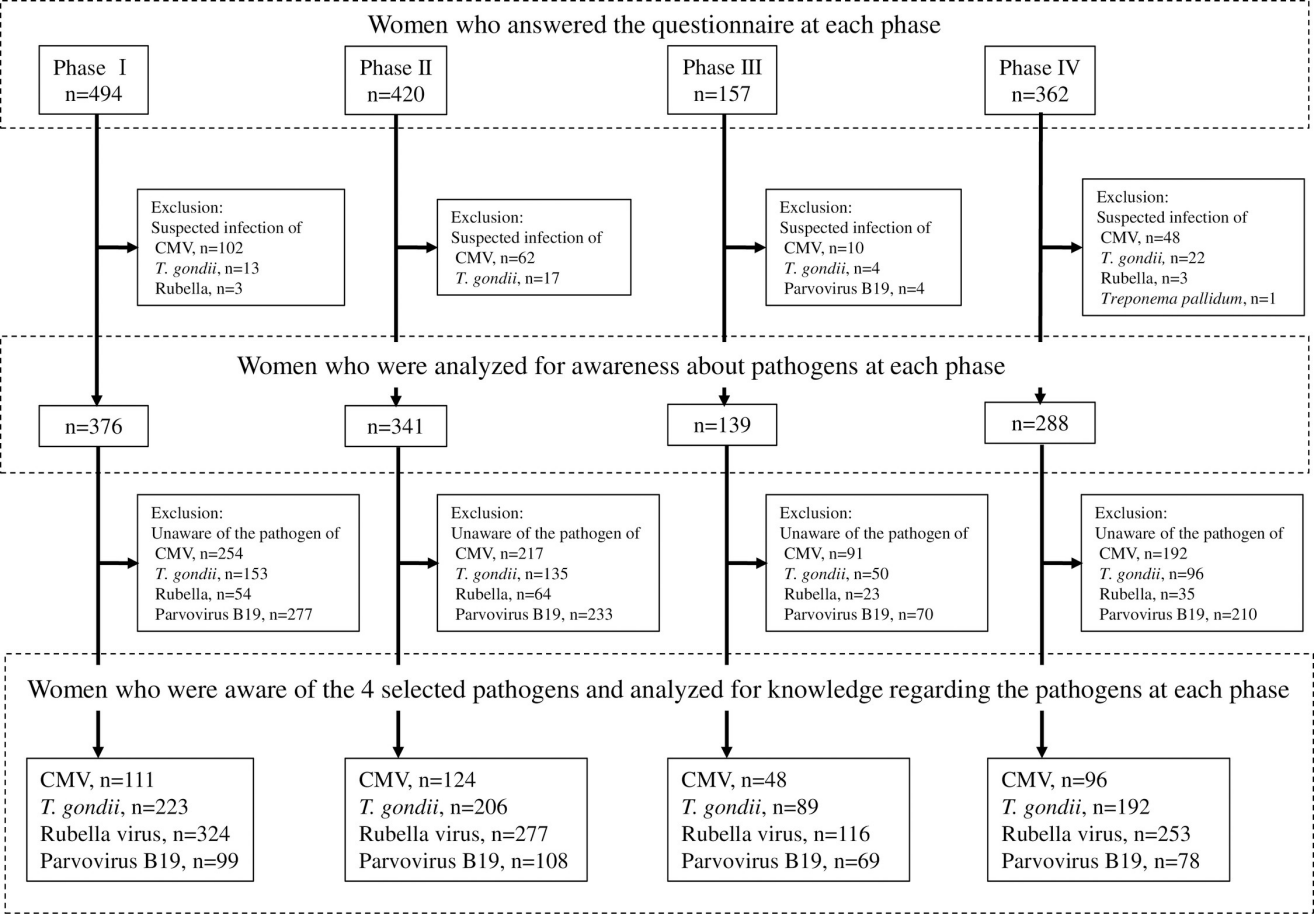
Patient flow diagram. CMV: Cytomegalovirus, *T*. *gondii*: *Toxoplasma gondii*.

The protocol of this survey was approved by the ethical committee of the Kobe University Graduate School of Medicine (approval number 1264). A questionnaire survey was conducted at the university hospital from October 2012 to January 2018. During this period, all pregnant women aged 18 years or older who gave written informed consent were invited to complete a written questionnaire regarding mother-to-child infections during their first visit to the outpatient clinics of their respective obstetrics department. Participation was voluntary, and the questionnaire was completed anonymously (Fig 1).

Since this questionnaire asked about awareness of and knowledge about mother-to-child infections, we excluded 289 pregnant women who were referred to our hospital for analysis due to suspicion of congenital infections. For detailed knowledge of the four selected pathogens, we excluded pregnant women who were unaware of each pathogen and only evaluated those who were aware of each pathogen.

### Contents of the questionnaire

A multiple-choice questionnaire assessed participants’ awareness of the following 13 pathogens of mother-to-child infections, which are identical to those used in our previous study [7]: CMV, *T*. *gondii*, hepatitis B virus, rubella virus, herpes simplex virus, parvovirus B19, hepatitis C virus, human immunodeficiency virus, human T cell leukemia virus type-1, measles virus, varicella-zoster virus, *Chlamydia trachomatis*, and *Treponema pallidum*. For the selected four pathogens (i.e., CMV, rubella virus, *T*. *gondii*, and parvovirus B19), the questionnaire also reviewed participants’ knowledge of transmission routes (i.e., a—droplets; b—children's urine and saliva, or semen; c—cat feces or eating undercooked meat; d—birth canal; e—breastmilk; f—I do not know), the most susceptible time of infection that may cause severe fetal diseases during pregnancy (i.e., a—before pregnancy; b—first trimester; c—second trimester; d—third trimester; e—I do not know), the maximum frequency of fetal infection in cases of maternal infection (i.e., a—[< 10%]; b—[10–50%]; c—[50–80%]; d—[> 80%], e—I do not know), and methods to prevent maternal infection (a—I know; b—I have ever heard; c—I do not know). The background characteristics of the participants, including age, occupation, history of childbirth and spontaneous miscarriage, gestational age at the time of the survey, and history of receiving a booklet on the prevention of infection during pregnancy, were also collected.

Correct answers for accurate knowledge of each pathogen were fully described in our previous publication [7].

### Statistical analysis

Data were expressed as the number (%) or median (range) of the subjects. The Chi-square test was used to compare the data of participants’ background, awareness, and knowledge about pathogens that can cause mother-to-child infections. Differences were deemed statistically significant at p<0.01. All statistical analyses were performed using the Excel statistical software package BellCurve for Excel, version 3.20, 2019 (Social Survey Research Information Co., Ltd., Tokyo, Japan).

## Results and discussion

### Characteristics of the participants

In total, 1433 pregnant Japanese women were included in this study. No pregnant women refused to participate during the study period. We divided the study periods into 4 phases constituting 16 months each, as phase I (October 2012-January 2014, n = 376), phase II (February 2014-May 2015, n = 341), phase III (June 2015-September 2016, n = 139), and phase IV (October 2016-January 2018, n = 288). Since this questionnaire asked about awareness of and knowledge about mother-to-child infections, we excluded 289 pregnant women who were referred to our hospital on suspicion of congenital infections (CMV; n = 220, *T*. *gondii*; n = 56, rubella virus; n = 6, parvovirus B19; n = 4, and *Treponema pallidum*; n = 1) from further analysis (Fig 1). The median age of the surveyed women was 34 years (range: 18 to 46 years), and among the 1144 women who were examined on their awareness of pathogens, 687 (60%) were primipara and 291 (25%) had previously experienced a spontaneous miscarriage. The median gestational age at the time of the survey was 15 weeks of gestation (range: 4 to 41). Altogether, 47 women (4%) were working as healthcare professionals (i.e., 24 nurses, 8 pharmacists, 6 nutritionists, 4 physiotherapists, 3 dental hygienists, and 2 doctors) and 30 as care workers (25 adult care workers and 5 childcare workers). There was no significant difference in the participants’ background among the phases (Table 1).

**Table 1:** Participants’ characteristics.

	Total n = 1144	Phase Ⅰ = 376	Phase Ⅱ n = 341	Phase Ⅲ n = 139	Phase Ⅳ n = 288	*P*-value
Age	34 (18–46)	35 (19–45)	33(18–44)	32 (18–45)	33 (18–46)	0.08
Primipara	687 (60.1%)	226 (60.1%)	193 (56.6%)	87 (62.6%)	181 (62.8%)	0.39
History of spontaneous miscarriage	291 (25.4%)	102 (27.1%)	89 (26.1%)	36 (25.9%)	64 (22.2%)	0.53
Gestational age	15 (4–41)	15 (4–40)	15 (4–41)	14 (4–38)	15 (4–41)	0.87
Healthcare professionals	47 (4.1%)	13 (3.4%)	18 (5.3%)	10 (7.0%)	6 (2.0%)	0.05
Care workers	25 (2.2%)	9 (2.4%)	8 (2.3%)	3 (2.1%)	5 (1.7%)	0.94
Received a booklet on infection prevention	3 (0.3%)	1 (0.3%)	1 (0.3%)	1 (0.7%)	0	0.59

*P-values* are shown for comparison among the four phases.

### Awareness of pathogens that can infect the fetus

**Fig 2:**
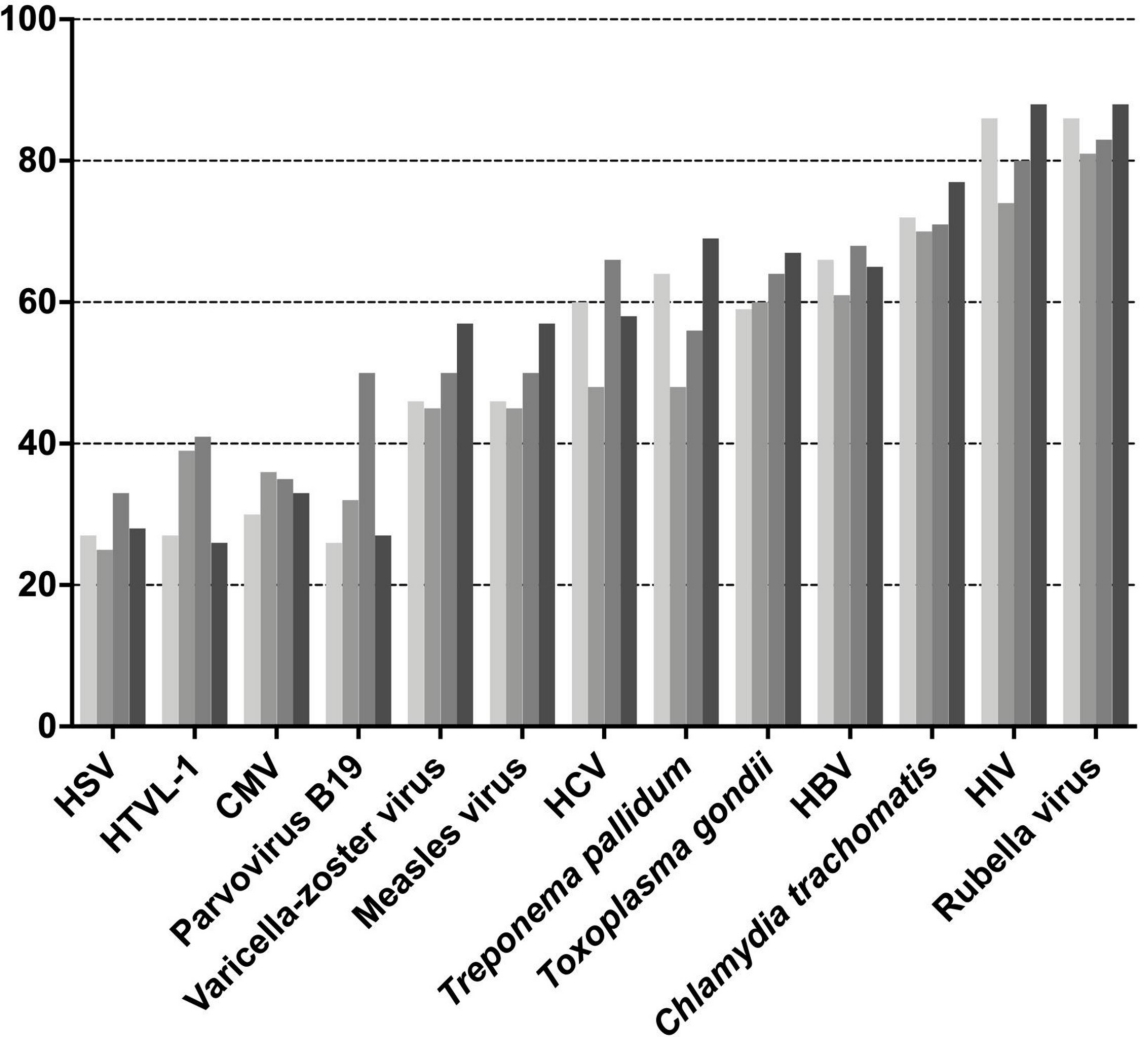
The proportion of pregnant women who are aware of various pathogens that can infect the fetus. HSV: Herpes simplex virus; HTLV-1: Human T-cell leukemia virus type 1; CMV: Cytomegalovirus; HCV: Hepatitis C virus; HBV: Hepatitis B virus; HIV: Human immunodeficiency virus.

The proportion of pregnant women who were aware of pathogens that can infect the fetus is shown in Fig 2.

The awareness of 8 out of 13 pathogens (i.e., HSV, CMV, VZV, measles virus, *T*. *gondii*, HBV, *Chlamydia trachomatis*, and rubella virus) did not differ between the periods. Significant changes in awareness were observed among the 5 pathogens (i.e., human T cell leukemia virus type-1, parvovirus B19, Hepatitis C virus, *Treponema pallidum*, and human immunodeficiency virus) across the periods; however, no pathogen exhibited a significant difference in its recognition between phase 1 and phase 4 (HTLV-1: 26.6% [phase 1] vs. 26.3% [phase 4]; parvovirus B19: 26.3% vs. 27.0%; HCV: 59.8% vs. 57.6%; *Treponema pallidum*: 64.1% vs. 68.8%; HIV: 85.9% vs. 87.8%, p > 0.2). During the total study period, the rubella virus was the most common pathogen (85%), followed by HIV (82%). Lastly, the proportions of women aware of pathogens associated with TORCH syndrome were as follows: rubella virus—85%, *T*. *gondii*—63%, *Treponema pallidum*—59%, parvovirus B19–33%, CMV—33%, and herpes simplex virus—28%.

### Awareness of rubella virus, *T*. *gondii*, parvovirus B19, and CMV, stratified by participants’ background characteristics

We stratified the awareness of rubella virus, *T*. *gondii*, parvovirus B19, and CMV by age category, history of childbirth, history of spontaneous miscarriage, gestational age category at the time of the survey, and employment as a healthcare professional or care worker (Table 2). In women with a history of childbirth, significantly higher awareness of CMV and Toxoplasma gondii (40.4% and 67.6%) was observed than in those without (28.3% and 58.3%, respectively, both for p<0.001). In women with a history of spontaneous abortion, a significantly higher awareness of Toxoplasma gondii was observed than in those without (70.5% vs. 59.1%, p<0.001). We also observed significantly higher awareness of CMV, *Toxoplasma gondii*, and parvovirus B19 in women working as healthcare professionals or care workers (59.6%, 80.9%, and 51.1%), compared to women not working in these positions (31.9%, 61.1%, and 30.0%, p<0.01, respectively).

**Table 2:** Participants’ characteristics.

	CMV		Rubella Virus		*Toxoplasma Gondii*		Parvovirus B19	
Characteristic	Aware	p	Aware	p	Aware	p	Aware	p
**Age**								
< 35 years	238/670 (35.5%)	0.04	554/670 (82.7%)	0.03	410/670 (61.2%)	0.53	198/670 (29.6%)	0.98
≥ 35 years	141/476 (29.6%)		416/476 (87.4%)		300/476 (63.0%)		141/476 (29.6%)	
**History of childbirth**								
Nullipara	197/695 (28.3%)	**<0.001**	574/695 (82.6%)	0.02	405/695 (58.3%)	**<0.001**	199/695 (28.6%)	0.04
Multipara	182/451 (40.4%)		396/451 (87.8%)		305/451 (67.6%)		155/451 (34.4%)	
**History of spontaneous miscarriage**								
≥ 1	108/285 (37.9%)	0.05	249/285 (87.4%)	0.30	201/285 (70.5%)	**<0.001**	99/285 (34.7%)	0.11
0	271/861 (31.5%)		721/861 (83.7%)		509/861 (59.1%)		255/861 (29.6%)	
**Gestational age at the time of the survey**								
< 22 weeks	283/839 (33.7%)	0.37	704/839 (83.9%)	0.47	519/839 (61.9%)	0.99	260/839 (31.0%)	0.90
≥ 22 weeks	95/307 (30.9%)		263/307 (85.7%)		190/307 (61.9%)		94/307 (30.6%)	
**Healthcare professional or care worker**								
Yes	28/47 (59.6%)	**<0.001**	45/47 (95.7%)	0.03	38/47 (80.9%)	**<0.01**	24/47 (51.1%)	**<0.01**
No	351/1,099 (31.9%)		925/1,099 (84.2%)		672/1,099 (61.1%)		330/1,099 (30.0%)	

*P-*values are shown for comparison between two groups.

In the following questionnaire regarding the four selected pathogens, we excluded pregnant women who were not aware of each pathogen, selecting only those women who were aware of each pathogen (Table 3).

**Table 3:** Detailed knowledge of four selected pathogens.

	Total	Phase Ⅰ	Phase Ⅱ	Phase Ⅲ	Phase Ⅳ	*P*-value
Rubella virus	n = 970	n = 324	n = 277	n = 116	n = 253	
Transmission	605 (62.4%)	239 (73.8%)	111 (40.1%)	91 (78.4%)	164 (64.8%)	**< 0.001**
Most susceptible time for severe fetal infection	520 (53.6%)	170 (52.5%)	114 (41.2%)	103 (88.8%)	133 (52.6%)	**< 0.001**
Maximum frequency of fetal infection	124 (12.8%)	41 (12.7%)	46 (16.6%)	8 (6.9%)	29 (11.5%)	0.05
Methods to prevent maternal infection	264 (27.2%)	95 (29.3%)	110 (39.7%)	14 (12.1%)	45 (17.8%)	**< 0.001**
***Toxoplasma Gondii***	n = 710	n = 223	n = 206	n = 89	n = 192	
Transmission	400 (56.3%)	154 (69.1%)	89 (43.2%)	31 (34.8%)	126 (65.6%)	**< 0.001**
Most susceptible time for severe fetal infection	273 (38.5%)	103 (46.2%)	55 (26.7%)	20 (22.5%)	95 (49.5%)	**< 0.001**
Maximum frequency of fetal infection	44 (6.2%)	13 (5.8%)	12 (5.8%)	7 (7.9%)	12 (6.3%)	0.91
Methods to prevent maternal infection	191 (26.9%)	57 (25.6%)	60 (29.1%)	12 (13.5%)	62 (32.3%)	**<0.01**
**CMV**	n = 379	n = 111	n = 124	n = 48	n = 96	
Transmission	156 (41.2%)	37 (33.3%)	67 (54%)	15 (31.3%)	37 (38.5%)	**<0.01**
Most susceptible time for severe fetal infection	83 (21.9%)	31 (27.9%)	24 (19.4%)	13 (27.1%)	28 (29.2%)	0.31
Maximum frequency of fetal infection	39 (10.3%)	16 (14.4%)	11 (8.9%)	0 (0%)	12 (12.5%)	0.04
Methods to prevent maternal infection	103 (27.2%)	25 (22.5%)	58 (46.8%)	13 (27.1%)	12 (12.5%)	**< 0.001**
**Parvovirus B19**	n = 354	n = 99	n = 108	n = 69	n = 78	
Transmission	80 (22.6%)	10 (10.1%)	33 (30.6%)	17 (24.6%)	20 (25.6%)	**<0.01**
Most susceptible time for severe fetal infection	21 (5.9%)	7 (7.1%)	5 (4.6%)	0 (0%)	9 (11.5%)	0.03
Maximum frequency of fetal infection	10 (2.8%)	4 (4%)	3 (2.8%)	0 (0%)	3 (3.8%)	0.42
Methods to prevent maternal infection	18 (5.1%)	7 (7.1%)	5 (4.6%)	5 (7.2%)	1 (1.3%)	0.23

*P-*values are shown for comparison among the four phases.

Regarding knowledge of transmission, all 4 pathogens revealed significant differences among the 4 study periods (p<0.01, respectively); nevertheless, no significant difference was found between phase I and phase IV for rubella virus (I: 73.8% vs. IV: 64.8%, p = 0.10), *Toxoplasma gondii* (I: 69.1% vs. IV: 65.6%, p = 0.46), or CMV (I: 33.3% vs. IV: 38.5%, p = 0.44). Only for parvovirus B19 was a significant increase found in phase IV (I: 10.1% vs. IV: 25.6%, p<0.01).

Concerning knowledge of the most susceptible time for severe fetal infection, there was no significant difference among the 4 study periods for CMV, and parvovirus B19, rubella, and *Toxoplasma gondii* presented significant differences in knowledge across study periods (p<0.001, respectively). However, no significant difference was found between phase I and phase IV for either rubella virus (I: 52.5% vs. IV: 52.6%, p = 0.98) or *Toxoplasma gondii* (I: 46.2% vs. IV: 49.5%, p = 0.50).

Regarding knowledge pertaining to the maximum frequency of fetal infection, there was no significant difference among the 4 study periods for all 4 pathogens.

Likewise, concerning knowledge of methods to prevent maternal infection, there was no significant difference among the 4 study periods for parvovirus B19. Contrariwise, rubella virus, *Toxoplasma gondii*, and CMV signified significant differences in knowledge among the study periods (p<0.01). Although no significant difference in knowledge between phases I and IV was found for *Toxoplasma gondii* (I: 25.6% vs. IV: 32.3%, p = 0.131) or CMV (I: 22.5% vs. IV: 12.5%, p = 0.06), a significant decrease in the knowledge of methods to prevent maternal infection was found for rubella virus in phase IV in comparison to phase I (I: 29.3% vs. IV: 17.8%, p<0.001).

Additionally, for all periods, knowledge about the transmission of rubella virus and *Toxoplasma gondii*, in tandem with the most susceptible time for the onset of a severe fetal infection of rubella virus, showed recognition rates of over 50% in pregnant women.

In the present study, there was no secular change in awareness of the pathogens over time through a questionnaire survey performed in a Japanese perinatal center between 2012 and 2018 regarding mother-to-child infections. We also verified that the detailed knowledge of the four pathogens of typical mother-to-child infections did not improve.

To the best of our knowledge, there have been few reports examining the secular change in knowledge about pathogens of mother-to-child infections. In the United States, Doutre et al. administered a mail-based questionnaire survey in all states using the US HealthStyles^TM^ to survey awareness related to five congenital infections (CMV, *Toxoplasma gondii*, rubella virus, parvovirus B19, HIV) in 2015 and 2016 (n = 4121 and 4197, respectively). Similar to our study, the awareness of each pathogen was consistently low (CMV: 7% [2015] and 7% [2016], *Toxoplasma gondii*: 8% and 9%, rubella virus: 17% and 13%, parvovirus B19; 23% and 20%, and HIV: 91% and 86%, respectively) and did not change over time [12]. Nonetheless, long-term secular changes were not examined in the two-year continuous survey. Additionally, the survey response rate (i.e., 67% in 2015 and 68% in 2016) was significantly lower than this study due to the employment of indirect mail-based questionnaire surveys.

Similar to a direct questionnaire survey with a high response rate (i.e., 92%, 643/700) like ours, Jeon et al. conducted a questionnaire survey in 7 US states in 2006, limited to women who visited obstetric clinics in 4 states and to the university staff and medical students in the remaining 3 states, and validated that awareness of congenital CMV infection was low (22%) as compared to parvovirus B19 (32%), congenital *Toxoplasmosis* (37%), and HIV (98%) [13]. The limitation of their study is that the survey target population is biased according to state in addition to utilizing a single-year survey. Further, in a questionnaire survey on awareness and knowledge of congenital CMV infection among healthcare providers in France, it was reported that awareness increased from 81% in 2012 to 95.8% in 2018. The authors considered the increased awareness to result from the systemized targeting of perinatal healthcare providers, such as obstetricians, pediatricians, and midwives, who had to inform pregnant women about congenital CMV infection as per the national guidelines [14]. We believe this is the first survey to investigate the changes in awareness and knowledge of various types of congenital infectious diseases over a prolonged duration and to be conducted in a single medical center with a uniform background and high response rate.

In a previous study, we reported that awareness of *Toxoplasma gondii* was significantly higher among healthcare workers than among non-healthcare workers (13/15 [87%] vs. 187/328 [57%]) [7]. In this study, all 4 pathogens of mother-to-child infections were highly recognized by healthcare workers, and awareness of CMV, *Toxoplasma gondii*, and parvovirus B19 was significantly higher. Compared to previous studies, we included more than 3 times the number of healthcare workers in this survey (n = 47), which might be reflected in the high awareness of congenital infections on the part of healthcare workers. Accordingly, our study depicts a correlation between awareness and detailed knowledge of 4 selected pathogens of mother-to-child infections. Still, less than 50% of people correctly deciphered the methods to prevent maternal infection against these 4 pathogens, and even healthcare workers demonstrated limited understanding. Since most pregnant women acquire knowledge from healthcare workers [15], we believe further educational interventions with healthcare workers are imperative. Further, in our previous study, there was no difference in awareness between primiparas and multiparous women [7]; however, in this study, multiparous women had a higher awareness of the pathogens than primiparas. Interestingly, it has been reported that primiparas are more likely to accept behavioral changes to prevent congenital cytomegalovirus [16]; thus, we believe that educational intervention with primiparas would be more effective. Moreover, pregnant women do undergo behavioral changes to protect their fetus, such as refraining from alcohol during pregnancy after a brief intervention [17]. In the same manner, providing correct knowledge to prevent mother-to-child infections could promote behavioral changes more readily.

This study has several limitations. First, the number of participants is not high compared to previously published reports; however, it still has substantial implications because of the sufficiently high response rate. Second, the survey was conducted in a single tertiary perinatal center; thus, it might not represent a general population of pregnant women in Japan.

## Conclusions

Throughout the 6-year survey, there was no change in the awareness of the pathogens that can cause mother-to-child infections over time, and no change was found in the detailed knowledge of the four selected pathogens. Additionally, since knowledge about methods to prevent maternal infection is still insufficient for all pathogens, active advocacy to prevent mother-to-child infections is needed.

## Supporting information

S1 File Eng request to participate in a questionnaire. (DOCX)

S2 File Japanese request to participate in a questionnaire. (DOCX)

## References

[1] Koyano S, Inoue N, Oka A, Moriuchi H, Asano K, Ito Y, et al Screening for congenital cytomegalovirus infection using newborn urine samples collected on filter paper: feasibility and outcomes from a multicentre study. BMJ Open. 2011;1(1): e000118 Epub 2011/10/25. 10.1136/bmjopen-2011-000118 22021766 PMC3191411

[2] Sasaki A, Sago H. Equipoise of recent estimated Down syndrome live births in Japan. Am J Med Genet A. 2019;179(9): 1815–1819. Epub 2019/07/18. 10.1002/ajmg.a.61298 . 31313514

[3] Yamada H, Tairaku S, Morioka I, Sonoyama A, Tanimura K, Deguchi M, et al Nationwide survey of mother-to-child infections in Japan. J Infect Chemother. 2015;21(3): 161–164. Epub 2014/12/02. 10.1016/j.jiac.2014.10.013 . 25435331

[4] Adler SP, Nigro G. Prevention of maternal-fetal transmission of cytomegalovirus. Clin Infect Dis. 2013;57 Suppl 4: S189–S192. Epub 2013/12/07. 10.1093/cid/cit585 . 24257425

[5] Revello MG, Tibaldi C, Masuelli G, Frisina V, Sacchi A, Furione M, et al Prevention of primary cytomegalovirus infection in pregnancy. EBioMedicine. 2015;2(9): 1205–1210. Epub 2015/10/27. 10.1016/j.ebiom.2015.08.003 26501119 PMC4588434

[6] Foulon W, Naessens A, Ho-Yen D. Prevention of congenital toxoplasmosis. J Perinat Med. 2000;28(5): 337–345. Epub 2000/12/29. 10.1515/JPM.2000.043 . 11125923

[7] Morioka I, Sonoyama A, Tairaku S, Ebina Y, Nagamata S, Morizane M, et al Awareness of and knowledge about mother-to-child infections in Japanese pregnant women. Congenit Anom (Kyoto). 2014;54(1): 35–40. Epub 2014/03/05. 10.1111/cga.12030 . 24588778

[8] Mori Y, Miyoshi M, Kikuchi M, Sekine M, Umezawa M, Saikusa M, et al Molecular epidemiology of rubella virus strains detected around the time of the 2012–2013 epidemic in Japan. Front Microbiol. 2017;8: 1513 Epub 2017/08/30. 10.3389/fmicb.2017.01513 28848523 PMC5553008

[9] Hori A, Wada K, Smith DR. A socio-demographic examination of adults responding to governmental vaccination recommendations during the Japanese rubella outbreak of 2013. PLoS One. 2015;10(6): e0129900 Epub 2015/06/10. 10.1371/journal.pone.0129900 26057740 PMC4461294

[10] Yoshikawa T, Kawamura Y, Ohashi M. Universal varicella vaccine immunization in Japan. Vaccine. 2016;34(16): 1965–1970. Epub 2016/03/06. 10.1016/j.vaccine.2016.02.058 . 26944711

[11] Ujiie M, Sasaki K, Yoshikawa N, Enami T, Shobayashi T. Introduction of a hepatitis B vaccine into the national routine immunisation programme of Japan. Lancet Infect Dis. 2016;16(12): 1325 Epub 2016/12/22. 10.1016/S1473-3099(16)30463-7 . 27998586

[12] Doutre SM, Barrett TS, Greenlee J, White KR. Losing ground: awareness of congenital cytomegalovirus in the United States. Journal of Early Hearing Detection and Intervention. 2016;1(2): 39–48. 10.15142/T32G62

[13] Jeon J, Victor M, Adler SP, Arwady A, Demmler G, Fowler K, et al Knowledge and awareness of congenital cytomegalovirus among women. Infect Dis Obstet Gynecol. 2006;2006: 80383 10.1155/IDOG/2006/80383 17485810 PMC1779612

[14] Fellah T, Sibiude J, Vauloup-Fellous C, Cordier AG, Guitton S, Grangeot-Keros L, et al Evolution of awareness and knowledge of congenital cytomegalovirus infection among health care providers in France between 2011 and 2018. J Clin Virol. 2020;129: 104335 Epub 2020/06/27. 10.1016/j.jcv.2020.104335 . 32590295

[15] Cordier AG, Guitton S, Vauloup-Fellous C, Grangeot-Keros L, Ayoubi JM, Benachi A, et al Awareness of cytomegalovirus infection among pregnant women in France. J Clin Virol. 2012;53(4): 332–337. Epub 2012/01/24. 10.1016/j.jcv.2011.12.031 . 22265828

[16] Tastad KJ, Schleiss MR, Lammert SM, Basta NE. Awareness of congenital cytomegalovirus and acceptance of maternal and newborn screening. PLoS One. 2019;14(8): e0221725 Epub 2019/08/27. 10.1371/journal.pone.0221725 PMC6709948 31449545

[17] Chang G, McNamara TK, Orav EJ, Koby D, Lavigne A, Ludman B, et al Brief intervention for prenatal alcohol use: a randomized trial. Obstet Gynecol. 2005;105(5 Pt 1): 991–998. Epub 2005/05/03. 10.1097/01.AOG.0000157109.05453.84 15863535 PMC1380262

