# Supplementary material for: Changes in awareness and knowledge concerning mother-to-child infections among Japanese pregnant women between 2012 and 2018
Source: PLoS One. 2021 Jan 6;16(1):e0244945. doi: 10.1371/journal.pone.0244945 (PMC7787470; doi:10.1371/journal.pone.0244945)
Supplement: S1 File — (DOCX) [file pone.0244945.s001.docx]

Request to participate in a questionnaire on mother-to-child transmission

Maternal infections during pregnancy can be passed from a mother to her fetus. Occasionally, this can cause congenital infections resulting in newborns exhibiting symptoms. To prevent this mother-to-child transmission, it is important that mothers take precautions to prevent infections during pregnancy. Kobe University Hospital is conducting a questionnaire survey as part of a study by the Ministry of Health, Labour and Welfare to establish a method to prevent mother-to-child transmission in Japan. Based on the results of the survey, we are aiming to prevent congenital infection in all obstetrics clinics in Japan and reduce the number of newborns with congenital infections.

　Thank you for your cooperation in this project.

1. Some infections can be harmful to the fetus if the mother gets infected for the first-time during pregnancy. Please choose any of the following microorganisms that you already know or have heard of. (You can choose multiple answers.)
2. *Toxoplasma gondii*
3. hepatitis B virus
4. rubella virus
5. cytomegalovirus
6. herpes simplex virus
7. parvovirus B19
8. hepatitis C virus
9. human immunodeﬁciency virus
10. human T cell leukemia virus type-1
11. measles virus
12. varicella-zoster virus
13. *Chlamydia trachomatis*
14. *Treponema pallidum.*

Below are some questions about prevention of such infections.

Please answer for each type of infection.

2) Which of the following is the route of transmission of "*Toxoplasma gondii*"?

1. droplets
2. semen or children’s urine and saliva
3. cat feces or eating undercooked meat
4. birth canal
5. breastmilk
6. I do not know

3) What is the most susceptible time during which "*Toxoplasma gondii*" may cause severe fetal diseases?

1. before pregnancy
2. first trimester
3. second trimester
4. third trimester
5. I do not know

4) What is the maximum frequency of fetal infection in cases of maternal infection with "*Toxoplasma gondii*" during pregnancy?

1. < 10%
2. 10-50%
3. 50-80%
4. > 80%
5. I do not know

5) Please select one of the following regarding prevention of "*Toxoplasma gondii*" infection.

1. I know how to prevent this infection
2. I have some idea about how to prevent this infection
3. I do not know how to prevent this infection

If you answered ① or ② for question 5), please answer question 6).

6) Please choose your prevention method below.

1. Wear a mask
2. Keep away from crowded places
3. Keep away from people with fever or rash
4. Wash hands and gargle
5. Wash hands after diaper changing
6. Avoid kissing a child under 6 years old
7. Avoid sharing food, drinks, or dishes and utensils with children
8. Avoid taking care of children under 2.5 years old if you are working in a daycare center
9. Use a condom during sexual intercourse
10. Avoid eating undercooked meat
11. Wear gloves during any contact with sand
12. Avoid trips to Europe during pregnancy
13. Keep away from cats
14. Get vaccinated before pregnancy
15. Delivery by Caesarean section
16. Avoid breastfeeding

7) Which of the following is the route of transmission of "rubella virus"?

1. droplets
2. semen or children’s urine and saliva
3. cat feces or eating undercooked meat
4. birth canal
5. breastmilk
6. I do not know

8) What is the most susceptible time during which "rubella virus" may cause severe fetal diseases?

1. before pregnancy
2. first trimester
3. second trimester
4. third trimester
5. I do not know

9) What is the maximum frequency of fetal infection in cases of maternal infection with "rubella virus" during pregnancy?

1. < 10%
2. 10-50%
3. 50-80%
4. > 80%
5. I do not know

10) Please select one of the following regarding prevention of "rubella virus" infection.

1. I know how to prevent this infection
2. I have some idea about how to prevent this infection
3. I do not know how to prevent this infection

If you answered ① or ② for question 10), please answer question 11).

11) Please choose your prevention method below.

1. Wear a mask
2. Keep away from crowded places
3. Keep away from people with fever or rash
4. Wash hands and gargle
5. Wash hands after diaper changing
6. Avoid kissing a child under 6 years old
7. Avoid sharing food, drinks, or dishes and utensils with children
8. Avoid taking care of children under 2.5 years old if you are working in a daycare center
9. Use a condom during sexual intercourse
10. Avoid eating undercooked meat
11. Wear gloves during any contact with sand
12. Avoid trips to Europe during pregnancy
13. Keep away from cats
14. Get vaccinated before pregnancy
15. Delivery by Caesarean section
16. Avoid breastfeeding

12) Which of the following is the route of transmission of "cytomegalovirus"?

1. droplets
2. semen or children’s urine and saliva
3. cat feces or eating undercooked meat
4. birth canal
5. breastmilk
6. I do not know

13) What is the most susceptible time during which "cytomegalovirus" may cause severe fetal diseases?

1. before pregnancy
2. first trimester
3. second trimester
4. third trimester
5. I do not know

14) What is the maximum frequency of fetal infection in cases of maternal infection with "cytomegalovirus" during pregnancy?

1. < 10%
2. 10-50%
3. 50-80%
4. > 80%
5. I do not know

15) Please select one of the following regarding prevention of "cytomegalovirus" infection.

1. I know how to prevent this infection
2. I have some idea about how to prevent this infection
3. I do not know how to prevent this infection

If you answered ① or ② for question 15), please answer question 16).

16) Please choose your prevention method below.

1. Wear a mask
2. Keep away from crowded places
3. Keep away from people with fever or rash
4. Wash hands and gargle
5. Wash hands after diaper changing
6. Avoid kissing a child under 6 years old
7. Avoid sharing food, drinks, or dishes and utensils with children
8. Avoid taking care of children under 2.5 years old if you are working in a daycare center
9. Use a condom during sexual intercourse
10. Avoid eating undercooked meat
11. Wear gloves during any contact with sand
12. Avoid trips to Europe during pregnancy
13. Keep away from cats
14. Get vaccinated before pregnancy
15. Delivery by Caesarean section
16. Avoid breastfeeding

17) Which of the following is the route of transmission of "parvovirus B19"?

1. droplets
2. semen or children’s urine and saliva
3. cat feces or eating undercooked meat
4. birth canal
5. breastmilk
6. I do not know

18) What is the most susceptible time during which "parvovirus B19" may cause severe fetal diseases?

1. before pregnancy
2. first trimester
3. second trimester
4. third trimester
5. I do not know

19) What is the maximum frequency of fetal infection in the case of maternal infection with "parvovirus B19" during pregnancy?

1. < 10%
2. 10-50%
3. 50-80%
4. > 80%
5. I do not know

20) Please select one of the following regarding prevention of "parvovirus B19" infection.

1. I know how to prevent this infection
2. I have some idea about how to prevent this infection
3. I do not know how to prevent this infection

If you answered ① or ② for question 20), please answer question 21).

21) Please choose your prevention method below.

1. Wear a mask
2. Keep away from crowded places
3. Keep away from people with fever or rash
4. Wash hands and gargle
5. Wash hands after diaper changing
6. Avoid kissing a child under 6 years old
7. Avoid sharing food, drinks, or dishes and utensils with children
8. Avoid taking care of children under 2.5 years old if you are working in a daycare center
9. Use a condom during sexual intercourse
10. Avoid eating under cooked meat
11. Wear gloves during any contact with sand
12. Avoid trips to Europe during pregnancy
13. Keep away from cats
14. Get vaccinated before pregnancy
15. Delivery by Caesarean section
16. Avoid breastfeeding

In the future, we will analyze the results of this survey and create and disseminate methods to prevent mother-to-child transmission in Japan.

Please also provide the following information if you would like to:

The date you filled out the questionnaire: / /

Your Age: years old

Pregnancy History: times

Delivery History: times

History of Spontaneous Abortion: times

Current weeks of pregnancy: weeks

Last Day of Menstruation: / /

Your Occupation:

Reasons for this visit:

Thank you for your cooperation.
