# Supplementary material for: Changes in awareness and knowledge concerning mother-to-child infections among Japanese pregnant women between 2012 and 2018
Source: PLoS One. 2021 Jan 6;16(1):e0244945. doi: 10.1371/journal.pone.0244945 (PMC7787470; doi:10.1371/journal.pone.0244945)
Supplement: S2 File — (DOCX) [file pone.0244945.s002.docx]

ウイルスなどの母子感染に関するアンケートのお願い

　妊娠中のウイルスなどの母体感染は、母親からお腹の赤ちゃんにうつることがあります。それによって、時に赤ちゃんが症状をもって生まれることがあります。そのようなことが起きないように、妊娠中の母親が感染しないように予防をすることが大切です。

神戸大学病院では、厚生労働省の研究の一環として、日本で役に立つ母子感染の予防法を作るためにアンケート調査を行っています。その結果をもとに、日本全国の産婦人科病院で母子感染の予防を行い、症状をもって生まれる赤ちゃんを日本で減少させることを目的としています。

　ご協力のほどを、よろしくお願いいたします。

以下に当てはるものに○を付けてください。

1. 妊娠中に母親が初めて感染すると、赤ちゃんに悪い影響がでることがある感染症があります。

以下の微生物のうち、すでに知っていたもの、または聞いたことがあるものに○を付けて下さい。（複数回答可）

1. トキソプラズマ
2. B型肝炎ウイルス
3. 風疹ウイルス
4. サイトメガロウイルス
5. 単純ヘルペスウイルス
6. パルボウイルス（リンゴ病）
7. C型肝炎ウイルス
8. HIVウイルス（エイズ）
9. ヒト成人T細胞性白血病ウイルス
10. 麻疹ウイルス
11. 水痘ウイルス
12. クラミジア
13. 梅毒

以下に、それぞれの感染予防についての質問があります。

下記の感染症について、お答え下さい。

2）「トキソプラズマ」の感染経路は以下のうちどれにあたるでしょうか？

1. 飛沫感染
2. 子供の唾液やし尿、精液や血液を介して
3. 猫などの糞便や生肉を介した経口感染
4. 産道感染
5. 母乳感染
6. 分からない

3)「トキソプラズマ」の感染で、どの時期が最も赤ちゃんに重い症状が出るでしょうか。

1. 妊娠前
2. 妊娠初期（妊娠１ヶ月〜４ヶ月）
3. 妊娠中期（妊娠５ヶ月〜７ヶ月）
4. 妊娠後期（妊娠８ヶ月以降）
5. 分からない

4）「トキソプラズマ」の妊娠中の初感染のうち、最大でおよそ何パーセントの赤ちゃんに感染をおこすでしょうか。

1. 10%未満
2. 10〜50%
3. 50〜80%
4. 80%以上
5. 分からない

5) 「トキソプラズマ」の感染予防の方法について

1. 知っていて日々心がけている
2. 聞いたことはある
3. 知らない

5)の質問で①,②と回答された方に、６）の質問に回答をお願いします。

6) 以下より、予防方法に当てはまると思うものに○をつけてください。（複数回答可）

1. マスクを着用する。
2. 人混みを避ける。
3. 発熱・発疹患者の近くに寄らない。
4. 手洗いやうがいをする。
5. オムツ（尿）、唾液接触後に15〜20秒の手洗いを励行する。
6. 6歳未満の子供の口や頬へキスをしない。
7. 子供と食べ物、飲み物、食器を共有しない。
8. 保育所などで働いている場合、2.5歳以上の子どもを担当する。
9. 性交渉時はコンドームを装着する。
10. 加熱不十分な肉やレバ刺し、ユッケ、生ハムを食べることを控える。
11. 手袋をして土いじりやガーデニングをする。
12. 妊娠中のヨーロッパ渡航は避ける。
13. 猫を避ける。
14. 非妊娠時にワクチンをうつ。
15. 帝王切開でお産をする。
16. 母乳をあげない。

7) 「風疹ウイルス」の感染経路は以下のうちどれにあたるでしょうか？

1. 飛沫感染
2. 子供の唾液やし尿、精液や血液を介して
3. 猫などの糞便を介して
4. 産道感染
5. 母乳感染
6. 分からない

8)「風疹ウイルス」の感染で、どの時期が最も赤ちゃんに重い症状がでるでしょうか。

1. 妊娠前
2. 妊娠初期（妊娠１ヶ月〜４ヶ月）
3. 妊娠中期（妊娠５ヶ月〜７ヶ月）
4. 妊娠後期（妊娠８ヶ月以降）
5. 分からない

9）「風疹ウイルス」の妊娠中の初感染のうち、最大でおよそ何パーセントの赤ちゃんに感染をおこすでしょうか。

1. 10%未満
2. 10〜50%
3. 50〜80%
4. 80%以上
5. 分からない

10) 「風疹ウイルス」の感染予防の方法について

1. 知っていて日々心がけている
2. 聞いたことはある
3. 知らない

10)の質問で①,②と回答された方に、11）の質問に回答をお願いします。

11) 以下より、予防方法に当てはまると思うものに○をつけてください。（複数回答可）

1. マスクを着用する。
2. 人混みを避ける。
3. 発熱・発疹患者の近くに寄らない。
4. 手洗いやうがいをする。
5. オムツ（尿）、唾液接触後に15〜20秒の手洗いを励行する。
6. 6歳未満の子供の口や頬へキスをしない。
7. 子供と食べ物、飲み物、食器を共有しない。
8. 保育所などで働いている場合、2.5歳以上の子どもを担当する。
9. 性交渉時はコンドームを装着する。
10. 加熱不十分な肉やレバ刺し、ユッケ、生ハムを食べることを控える。
11. 手袋をして土いじりやガーデニングをする。
12. 妊娠中のヨーロッパ渡航は避ける。
13. 猫を避ける。
14. 非妊娠時にワクチンをうつ。
15. 帝王切開でお産をする。
16. 母乳をあげない。

12) 「サイトメガロウイルス」の感染経路は以下のうちどれにあたるでしょうか？

1. 飛沫感染
2. 子供の唾液やし尿、精液や血液を介して
3. 猫などの糞便を介して
4. 産道感染
5. 母乳感染
6. 分からない

13)「サイトメガロウイルス」の感染で、どの時期が最も赤ちゃんに重い症状がでるでしょうか。

1. 妊娠前
2. 妊娠初期（妊娠１ヶ月〜４ヶ月）
3. 妊娠中期（妊娠５ヶ月〜７ヶ月）
4. 妊娠後期（妊娠８ヶ月以降）
5. 分からない

14）「サイトメガロウイルス」の妊娠中の初感染のうち、最大でおよそ何パーセントの赤ちゃんに感染をおこすでしょうか。

1. 10%未満
2. 10〜50%
3. 50〜80%
4. 80%以上
5. 分からない

15) 「サイトメガロウイルス」の感染予防の方法について

1. 知っていて日々心がけている
2. 聞いたことはある
3. 知らない

15)の質問で①,②と回答された方に、16）の質問に回答をお願いします。

16) 以下より、予防方法に当てはまると思うものに○をつけてください。（複数回答可）

1. マスクを着用する。
2. 人混みを避ける。
3. 発熱・発疹患者の近くに寄らない。
4. 手洗いやうがいをする。
5. オムツ（尿）、唾液接触後に15〜20秒の手洗いを励行する。
6. 6歳未満の子供の口や頬へキスをしない。
7. 子供と食べ物、飲み物、食器を共有しない。
8. 保育所などで働いている場合、2.5歳以上の子どもを担当する。
9. 性交渉時はコンドームを装着する。
10. 加熱不十分な肉やレバ刺し、ユッケ、生ハムを食べることを控える。
11. 手袋をして土いじりやガーデニングをする。
12. 妊娠中のヨーロッパ渡航は避ける。
13. 猫を避ける。
14. 非妊娠時にワクチンをうつ。
15. 帝王切開でお産をする。
16. 母乳をあげない。

17) 「パルボウイルス」の感染経路は以下のうちどれにあたるでしょうか？

1. 飛沫感染
2. 子供の唾液やし尿、精液や血液を介して
3. 猫などの糞便を介して
4. 産道感染
5. 母乳感染
6. 分からない

18)「パルボウイルス」の感染で、どの時期が最も赤ちゃんに重い症状が出るでしょうか。

1. 妊娠前
2. 妊娠初期（妊娠１ヶ月〜４ヶ月）
3. 妊娠中期（妊娠５ヶ月〜７ヶ月）
4. 妊娠後期（妊娠８ヶ月以降）
5. 分からない

19）「パルボウイルス」の妊娠中の初感染のうち、最大でおよそ何パーセントの赤ちゃんに感染をおこすでしょうか。

1. 10%未満
2. 10〜50%
3. 50〜80%
4. 80%以上
5. 分からない

20) 「パルボウイルス」の感染予防の方法について

1. 知っていて日々心がけている
2. 聞いたことはある
3. 知らない

20)の質問で①,②と回答された方に、21）の質問に回答をお願いします。

21) 以下より、予防方法に当てはまると思うものに○をつけてください。（複数回答可）

1. マスクを着用する。
2. 人混みを避ける。
3. 発熱・発疹患者の近くに寄らない。
4. 手洗いやうがいをする。
5. オムツ（尿）、唾液接触後に15〜20秒の手洗いを励行する。
6. 6歳未満の子供の口や頬へキスをしない。
7. 子供と食べ物、飲み物、食器を共有しない。
8. 保育所などで働いている場合、2.5歳以上の子どもを担当する。
9. 性交渉時はコンドームを装着する。
10. 加熱不十分な肉やレバ刺し、ユッケ、生ハムを食べることを控える。
11. 手袋をして土いじりやガーデニングをする。
12. 妊娠中のヨーロッパ渡航は避ける。
13. 猫を避ける。
14. 非妊娠時にワクチンをうつ。
15. 帝王切開でお産をする。
16. 母乳をあげない。

今後、この調査結果を分析して、日本の母子感染の予防方法を作り広めていきます。そのために、必要な情報として、よろしければ以下の記載もお願いいたします。

アンケート記載年月日　H　　年　　月　　日

御年齢　　　　歳

妊娠歴　　　　回（今回は含まない）

分娩歴　　　　回

自然流産歴　　　回

妊娠されている方は、妊娠およそ　　　週

最終月経日　　　月　　日　〜　　月　　日

御職業

今回受診された理由

ご協力ありがとうございました。
